# Accounting for Movement Increases Sensitivity in Detecting Brain Activity in Parkinson's Disease

**DOI:** 10.1371/journal.pone.0036271

**Published:** 2012-05-01

**Authors:** Štefan Holiga, Harald E. Möller, Tomáš Sieger, Matthias L. Schroeter, Robert Jech, Karsten Mueller

**Affiliations:** 1 Max Planck Institute for Human Cognitive and Brain Sciences, Leipzig, Germany; 2 Department of Neurology and Center of Clinical Neuroscience, Charles University in Prague, Prague, Czech Republic; 3 Department of Cybernetics, Czech Technical University in Prague, Prague, Czech Republic; 4 Clinic for Cognitive Neurology and Leipzig Research Center for Civilization Diseases, University of Leipzig, and FTLD Consortium, Leipzig, Germany; University Medical Center Groningen UMCG, The Netherlands

## Abstract

Parkinson's disease (PD) is manifested by motor impairment, which may impede the ability to accurately perform motor tasks during functional magnetic resonance imaging (fMRI). Both temporal and amplitude deviations of movement performance affect the blood oxygenation level-dependent (BOLD) response. We present a general approach for assessing PD patients' movement control employing simultaneously recorded fMRI time series and behavioral data of the patients' kinematics using MR-compatible gloves. Twelve male patients with advanced PD were examined with fMRI at 1.5T during epoch-based visually paced finger tapping. MR-compatible gloves were utilized online to quantify motor outcome in two conditions with or without dopaminergic medication. Modeling of individual-level brain activity included *(i)* a predictor consisting of a condition-specific, constant-amplitude boxcar function convolved with the canonical hemodynamic response function (HRF) as commonly used in fMRI statistics (standard model), or *(ii)* a custom-made predictor computed from glove time series convolved with the HRF (kinematic model). Factorial statistics yielded a parametric map for each modeling technique, showing the medication effect on the group level. Patients showed bilateral response to levodopa in putamen and globus pallidus during the motor experiment. Interestingly, kinematic modeling produced significantly higher activation in terms of both the extent and amplitude of activity. Our results appear to account for movement performance in fMRI motor experiments with PD and increase sensitivity in detecting brain response to levodopa. We strongly advocate quantitatively controlling for motor performance to reach more reliable and robust analyses in fMRI with PD patients.

## Introduction

Parkinson's disease (PD) is a progressive neurodegenerative disorder causing basal ganglia (BG) dysfunction [1]. It is characterized by a large number of motor and non-motor deficits, which significantly contribute to reduced quality of life. Despite the definition of the broad spectrum of clinical characteristics and criteria for diagnostics [2], mechanisms triggering illness, the nature of its progression, and the character of therapeutic effects are still a matter of debate [1], [3]–[6]. Motor symptoms are key features of clinical criteria and are essential for the diagnosis of PD and its differentiation from related disorders. Bradykinesia, tremor, rigidity, and postural instability are regarded as cardinal symptoms and are associated with difficulties with planning, initiating and executing movements, performing sequential and simultaneous tasks, progressively reduced magnitude of sequential movements, involuntary choreatic and dystonic movements, hesitation in initiation, or finishing voluntary movements [2].

For the last two decades, positron emission tomography (PET) and functional magnetic resonance imaging (fMRI) have been used to investigate the neural substrates of motor deficits in PD [7], [8]. More recently, fMRI studies have tended to outnumber PET studies due to their advantages of greater temporal and spatial resolution and non-invasiveness [8]. Frequently used block-design paradigms primarily utilize the upper limbs, in particular, various hand and finger movement sequences, to investigate the neural basis of PD patients' motor performance. This is motivated by a higher degree in limitations of potential movement complexity and larger hand cortical representation [9]. Prevalent tasks in investigating the brain motor circuitry in PD are sequential finger movements, which are also part of the widely used Unified PD Rating Scale (UPDRS) [10]. This clinical scoring system rates the symptomatic severity of the disease. It is easily accessible and suitable for providing a direct comparison between subjects or use in longitudinal studies.

To assess the correctness of task execution and confirm comparable performance among PD participants, previous imaging studies have used push buttons [11]–[13], video-camera recordings [14]–[16], observers/raters [17], custom-built systems [18], or no specific arrangements [19]. Prior training sessions have also been employed [15], [17], [20]–[23] to practice the task and obtain adequate performance. A recent study [24] assessed the motor outcome of PD patients quantitatively using electromyography (EMG) and fMRI. In particular, measured fluctuations of the tremor amplitude were used to identify tremor-related brain activity. However, no study has explicitly considered the effect of accounting for the motor outcome on the sensitivity in detecting task-related brain activity in fMRI experiments with PD patients so far.

The analysis and interpretation of task-dependent fMRI data is highly dependent on the choice of the hemodynamic response (HR) model. Hemodynamic timing variability limits the interpretation of fMRI data because of its relatively rapid time scale ranging from milliseconds to seconds [25]. Accurate modeling of motor-related brain responses in fMRI investigations therefore relies on the experimental timing to a great extent. Another important characteristic is the movement amplitude. Large-amplitude movements seem to lead to an increased blood-oxygenation level dependent (BOLD) response in several brain regions as compared to small-amplitude movements [26]. Other factors influencing the HR include force [27], reaction time [28], and movement rate [29], [30]. Due to this high sensitivity of the BOLD response, modeling of fMRI data should ideally consider all potential factors affecting the shape or timing. The fine-grained nature of the BOLD signal is further underlined by its relatively tenuous changes of ∼1% in BG [31], regions that are predominantly affected by PD. In investigations of PD subjects, this becomes even more apparent due to motion variability because of bradykinesia and hypokinesa, impaired initiation of the movements after internal or sensory cues, frequent hesitation or freezing of the movements, resting tremor and dyskinesias while performing motor tasks. The motor abnormalities in PD are not always easily differentiable and may even manifest as a mixture of several symptoms with a great degree of variability between patients. Unfortunately, all this may compromise the correct interpretation of the functional imaging of motor tasks in general. In investigations of therapeutic effects, for example medication or deep brain stimulation, requiring repeated fMRI sessions and where the motor outcome of participants may even dramatically change between the sessions intra-individually, experimental accuracy and reproducibility is a critical issue. Therefore, it is impossible to achieve favorable accuracy in modeling the BOLD response without explicitly and quantitatively assessing participants' motor outcomes. Without such knowledge, statistical tests relying on the standard BOLD model will be degraded by inappropriate estimates of partial regression coefficients, *ß*, and thus result in biased, unreliable, and potentially invalid conclusions. The goals of the current study were to confirm this statement and present a robust solution to problems arising from it. To emphasize the possibility of within-subject, but also between-subject, task-related deviations caused by the broad motor heterogeneity of the disease, we designed the study with repeated measurement sessions, by employing experimental manipulation (levodopa medication) having a radical, and more importantly, individual task-dependent effect on the response of the participants. With this experimental setup we hypothesized that in contrast to generic fMRI statistics, accounting for deviations in task execution quantitatively in BOLD modeling improves the accuracy of detecting individual motor activity by reducing ‘type II’ errors. Consequently, this should result in increased sensitivity of detecting the patients' brain responses to levodopa medication on the group level.

Several attempts to record kinematic information on-line during motor tasks for consideration in hemodynamic modeling have already been proposed. Most prevalent and established techniques employ simultaneous fMRI and EMG recordings in healthy subjects to validate brain activation by their relation to the EMG recordings [32]–[35]. An optoelectronic motion capture system monitoring a stroke patient [36] or a custom-built sensing system [37] have also been utilized in clinical studies. Recently, instrumented, MRI-compatible gloves for capturing movements during fMRI investigations have been introduced. Specifically, gloves were used in investigations of correlates of finger movements and brain activity [38] or for qualitative control in a study in stroke patients [39]. In the current work, the gloves were employed during simultaneous recordings of fMRI and kinematics in serial investigations of PD patients to evaluate potential improvements in the statistical data analysis.

## Materials and Methods

### Patients

Twelve right-handed male patients with advanced akinetic-rigid type of PD (Hoehn-Yahr stages II–III, 45–64 years of age) [40] were recruited for this study. As basis for the diagnosis, the UK PD Society Brain Bank Criteria [41] were used. All patients included in the study met the criteria. Each of them gave written informed consent prior to participation in accordance with the declaration of Helsinki. Ethics Committee of the General University Hospital in Prague, Czech Republic approved the protocol of the study. Severity of patients' motor symptoms was clinically assessed using the motor examination (part III) of the UPDRS. UPDRS-III score sheets were used to evaluate hemibody scores comprising information about dominant lateral involvement of PD by summing rigidity (sum of item 22), akinesia (sum of items 19, 23–26, 31), and tremor (sum of items 20, 21) for each hemibody separately [10], [42]. Patients' clinical and demographic characteristics are summarized in Table 1. For detailed individual information see Table S1.

**Table 1:** Demographic and clinical summary of studied patients (N = 12).

Characteristic	Mean (SD)	Range
Age (years)	56.0 (7.0)	45–64
Gender (M/F)	12/0	-
Disease duration (years)	12.4 (2.0)	9–15
Levodopa treatment duration (years)	9.3 (3.0)	5–13
Motor complications duration (years)	5.0 (3.0)	2–12
UPDRS^*^-III: levodopa OFF	33.5 (9.0)	20.5–47.0
UPDRS^*^-III: levodopa ON	9.6 (4.0)	1.5–20.5
MMSE^†^	28.9 (1.0)	28–30

UPDRS* - Unified Parkinson's Disease Rating Scale. MMSE^†^ - Mini Mental State Examination.

Patients were measured in two conditions, once after overnight withdrawal of levodopa (‘levodopa OFF’ condition) and once one hour after administration of 250 mg of levodopa/25 mg carbidopa (‘levodopa ON’ condition) (Isicom 250, Desitin Arzneimittel, Hamburg, Germany). Any other anti-parkinson's medication (dopamine agonists, selegiline, amantadine, anticholinergics) was not administered for four days before the medication-free condition measurement.

### MRI data acquisition

Functional imaging was performed on a 1.5T MAGNETOM Symphony scanner (Siemens, Erlangen, Germany) using a birdcage head coil. A *T*_2_*-weighted gradient-echo echo-planar imaging (EPI) sequence (flip angle 90°; repetition time, *TR* = 1 s; echo time, *TE* = 54 ms) was used for BOLD fMRI. Ten oblique slices (thickness 3 mm; 1-mm slice separation; nominal in-plane resolution 3×3 mm^2^) were acquired. The slices were oriented along the central sulcus, covering the primary sensorimotor cortex and the basal ganglia. Additionally, a three-dimensional *T*_1_-weighted dataset was acquired with a magnetization prepared rapid acquisition gradient echo (MP-RAGE) sequence in 160 axial slices 1.65-mm thick with nominal in-plane resolution 0.9×0.9 mm^2^ and field of view (FOV) 238 mm covering the entire brain and cerebellum (inversion time, *TI* = 1100 ms; *TR* = 2140 ms; flip angle 15°; *TE* = 3.93 ms) for registration and display of fMRI results.

### Movement monitoring set-up

Patients' motor outcome was recorded using instrumented, MRI-compatible, bilateral sensory gloves (5^th^ Dimension Technologies, Irvine, CA, USA). The glove contains no ferromagnetic parts and communicates with a control box placed outside the scanner room via optical cable. The control box is connected with a remote computer via USB or serial port. The glove is made of a stretch lycra material (fits to many hand sizes) with embedded proprietary fiber-optic-based flexor technology sensors. Two sensors per finger measure flexion of its knuckle and first joint. One sensor quantifies the abduction between particular fingers. A set of 14 sensors allows the complexity of various finger movement patterns or gestures to be captured with a maximum sampling rate of 100 Hz and amplitude resolution of 8 data bits. The gloves were linked to an in-house-built EVSENG system (J. Wackermann, T. Sieger) for synchronization with the MRI scanner and on-line recording of the information from the glove. EVSENG was written to communicate with the glove on the low-level (i.e. reading data directly from port), but a high-level interface is also possible via libraries and routines supported by the producer.

### Experimental paradigm

A block-based motor paradigm was conceived to investigate the brain activity associated with the motor performance. Consecutive movement and rest epochs, each lasting 10 s, recurred 25 times, resulting in 50 blocks with a total session length of 500 s. During rest epochs, a visual ‘rest signal’ (centered static red fixation cross on a black background) was presented on a projection screen, whereas during movement epochs, 10 pacing ‘movement cues’ (yellow square behind the fixation cross displayed for 100 ms) were presented with a frequency of 1 Hz. While viewing the ‘rest signal’, patients were instructed to retain motionless with their arms in a resting position. During movement epochs, patients had to perform a unilateral index finger-thumb opposition whenever the ‘movement signal’ appeared. For ideal performance, a session would consist of a total of 250 distinct unilateral movements. The first measurement session started with right-hand movements and was subsequently repeated for the contralateral hand in the particular medication condition. Prior to the fMRI experiment, patients had to perform a calibration gesture (fully clenched fist followed by one index finger-thumb opposition) to allow the flexor sensors to reach their peak values and accommodate the amplitude dynamic range.

A two-by-two factorial design with within-subject factors ‘Hand’ (RIGHT/LEFT) and ‘Levodopa medication’ (OFF/ON) resulted in four scanning sessions for each patient.

### Glove recording processing

For each session, a 14-dimensional kinematic signal was recorded with the glove with a sampling rate of 64 Hz. Processing was performed using Matlab® (R2010b, The MathWorks Inc., Natick, MA, USA) and subroutines of the SPM8 package (Wellcome Trust Centre for Neuroimaging, UCL, London, UK).

To consider potential inter/intra-individual differences in the dynamic range of the finger movements, a normalization procedure was conducted to obtain consistent scaling of the signal amplitude. The calibration sequence was used to detect peak and baseline of the movement, and the signal was then normalized accordingly by adjusting the peak amplitude to one. Removal of low frequency fluctuations and drifts was achieved by subtracting the output of a fast one-dimensional median filter [43] with a 20-s window from the original signal. Substantial amount of high frequency quantization noise was primarily present in signals recorded from less active sensors with the restrained dynamic range. Wavelet-based de-noising [44] was applied in order to remove the noise using the global thresholding and a ‘db3’ wavelet filter family.

Glove waveforms were first analyzed independently on a behavioral level. Data-driven filtering using principal component analysis (PCA) [45] was performed to tease apart the global/deterministic and residual/stochastic features of movement. For a comprehensive description of data-driven filtering using PCA, see Daffertshofer et al. [46]. The global pattern represented a coherent, dominant pattern (the finger tapping movement itself) and was calculated for each session by reconstructing the 14-dimensional dataset with principal components explaining more than 90% of the data. Remaining principal components were used to reconstruct the residual part of the data. The residual part reflected movement deviations in participants' performance. Variance of each filtered waveform was calculated and averaged across sensors to obtain the average variance of a session for both the global and residual movement pattern. The variances of a session were separated and averaged across particular levels of experimental factors (RIGHT, LEFT, OFF, ON) and statistically analyzed inter-individually using analysis of variance with repeated measures (rmANOVA) with IBM SPSS Statistics 19.

Pre-processed glove recordings were first synchronized with the timing of MR images acquisition. To build a personalized regressor as input to the individual-level fMRI design matrix, time-courses from 14 glove sensors in a session were merged using two distinct approaches based on linear Gaussian models. *(i)* The ‘mean approach’ resulted in a waveform computed from the average of all 14 session-specific waveforms. *(ii)* The ‘eigenvariate approach’ reflected the session-specific movement recordings in terms of the projection of glove data on the first principal component, which explained the highest proportion of variance of the input observations. This calculation was based on PCA. Both mean and eigenvariate versions of waveforms were corrected for outliers by replacing them with maximal/minimal values in the non-outliers range. Outliers were defined as data points, which were more than 1.5 times the interquartile range above the third or below the first quartile. Frequency spectra of all waveforms were observed, to verify the absence of any restlessness and rhythmic motions in frequency band of 4–7 Hz (i.e. resting tremor/dyskinesias) during resting phase of the task. Furthermore, when no significant peaks of movement performance were detected within data values of resting periods, they were adjusted to zero. Besides spectral analysis of the resting periods we also focused on motor periods of the task. No peaks suggesting presence of low (4–6 Hz), intermediate (6–8 Hz) or higher (8–20 Hz) frequency of tremor in any patient were observed. Finally, for both approaches the envelope curve of the resulting waveform was calculated. To investigate the effect of movement amplitude on the brain response, amplitude-invariant versions of the mean and eigenvariate predictors were additionally formed by adjusting amplitudes of the waveforms in movement periods to unity. This adjustment provided time series sensitive to the timing of the movement execution, but invariant in terms of the amplitude of movement performance. Finally, signals were resampled to match the number of acquired fMRI time points. Four distinct waveforms – mean; mean amplitude-invariant (AI); eigenvariate; eigenvariate AI – were constructed for each participant this way and used in further analyses in SPM.

### fMRI time series analyses

Data pre-processing and analysis was performed using SPM8 in Matlab® for every session separately. To correct for head movement artifacts, fMRI data were spatially realigned to the first image. The individual *T*_1_-weighted MP-RAGE dataset was co-registered with the functional images, segmented with the unified segmentation approach (UnSA) [47] and normalized to the Montreal Neurological Institute (MNI) [48] *T*_1_ template. Normalization parameters from UnSA were then used to normalize all remaining images. In the final stage of pre-processing, the functional volumes were smoothed using an isotropic Gaussian kernel of 8-mm full width at half maximum [49].

Fixed-effects, first-level statistics were performed using a general linear model (GLM). Two separate types of models were used: (i) a standard model incorporating a constant baseline term and a predictor containing a condition-specific, constant-amplitude boxcar function characterized by onsets and durations of task-related epochs. This generic model was generated by a conventional procedure standard to SPM that assumed movements coinciding precisely with cues presented on-screen. (ii) a kinematic model including the constant term and a custom-made predictor – one possible alternative to mean, eigenvariate or their amplitude-invariant/amplitude-sensitive approaches, calculated from kinematic recordings as described in ‘Glove recording processing’.

**Figure 1:**
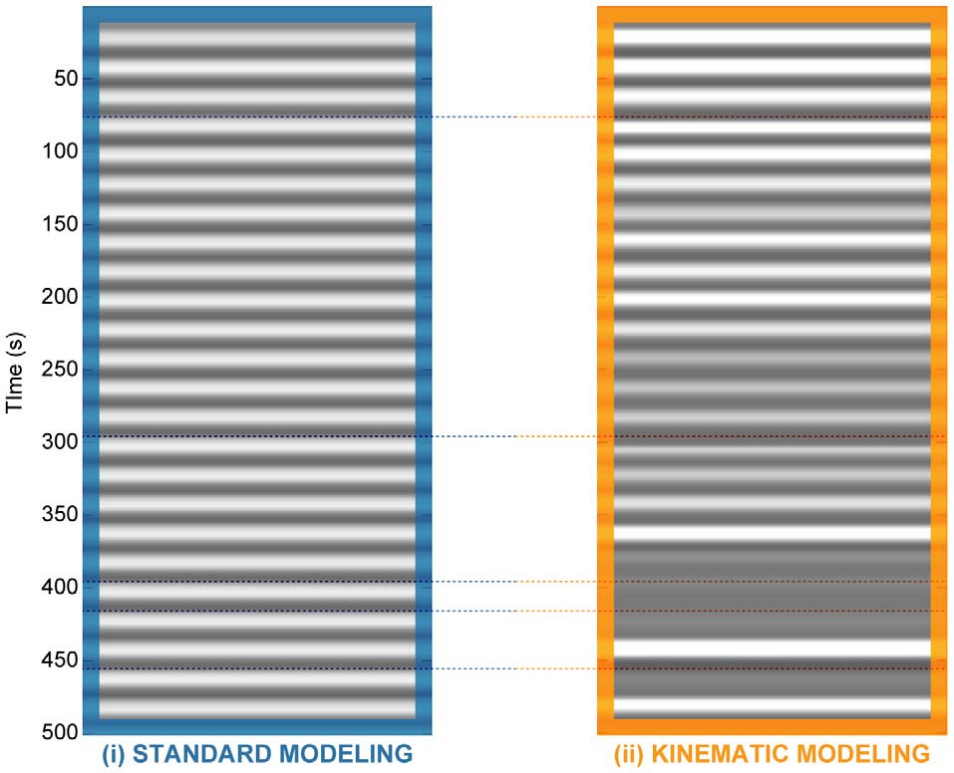
Comparison of session-specific predictors of standard and kinematic approaches in individual-level modeling. (left, blue: standard approach, generated using experimental timing information and no movement assumptions; right, orange: kinematic, mean amplitude-sensitive approach, constructed using average of recorded kinematics from all sensors). Dashed lines indicate the most pronounced movement deviations in a measurement session of a particular patient. Standard modeling is not able to capture this variability and reflects it in the error term in GLM, likely resulting in biased statistics.

Further processing was conventional and common to both the standard and kinematic approaches. It involved high-pass filtering of a 32-s cutoff for removing the most possible amount of low-frequency fluctuations while sustaining no loss of experimental power, first-order autoregressive *AR*(1) model [50], [51] for estimating the intrinsic correlations between residual errors, and a linear time-invariant (LTI) convolution model [52] based on a linear approximation of the BOLD response. The session-specific predictor of each modeling approach was convolved with a canonical hemodynamic response function (HRF) modeled as the first-order Volterra kernel [53], [54]. Parameters of Gaussians modeling were determined by timing characteristics of the experiment. Since amplitude-sensitive varieties of kinematic predictors had no baseline-to-peak unique range, they were additionally standardized by scaling the values (5th to 95th percentile) range of each to unity to consequently ensure valid comparisons of *ß*-estimates between different modeling approaches. Fixed-effects analysis was performed by fitting the mass-univariate GLM to calculate parameter estimates and residual errors. The standard model and four distinct kinematic models were estimated for each patient and session. Figure 1 illustrates standard and one of the kinematic predictors for a particular patient's session. Contrasting the effect of interest (non-constant session-specific regressor of the design matrix) resulted in contrast images which were used as input for random-effects group analyses.

To evaluate the effect of dopaminergic medication on brain correlates of finger motion in the group of PD patients investigated, both medication conditions were studied separately for each hand by estimating one-sample *t*-test random-effects models. Four models for each level of experimental factor were estimated for each (standard, kinematic) modeling approach. The group maps were thresholded at an uncorrected rate of *p*<0.001, with the threshold extended to 150 voxels to preserve only clusters corrected for multiple comparisons using the family-wise error (FWE) [55] rate of *p*<0.05 at the peak level. The amplitude of activity was inspected in regions of interest (ROI) in the left and right precentral gyrus. The ROIs were generated using automated anatomical labeling (AAL) atlas [56] with Marsbar SPM toolbox [57]. Proper scaling of predictors (unit values) and setting contrast levels (absolute sum of unity) in individual-level models ensured their maximal interpretability and comparability; the contrast images calculated approximated the percent signal change (PSC) directly [58]. We formed the ‘group-level mean’ and ‘group-level standard error’ PSC image for each levodopa medication condition and extracted the values from ROI and displayed their average for every modeling approach, for every level of experimental factors separately. In order to take inter-individual differences into account, PSC values were extracted and averaged from the ROI inter-individually. IBM SPSS Statistics was then used to calculate rmANOVA with factors ‘Hand’ and ‘Modeling approach’ for both levodopa ON and levodopa OFF medication conditions. Hence, the standard approach was compared to each particular kinematic approach in a pairwise fashion. Using the same technique, differences between particular (mean, eigenvariate) amplitude-invariant and amplitude-sensitive kinematic approaches were evaluated.

The difference between medication conditions was assessed using a flexible-factorial model with within-subjects factors ‘Hand’ (RIGHT/LEFT) and ‘Levodopa medication’ (OFF/ON) by choosing the difference between the ON and OFF conditions as an effect of interest. The analyses were carried out for both standard and kinematic approaches. An uncorrected threshold of *p*<0.001 with 30 voxels extent was adopted. On the cluster level, an FWE rate of *p*<0.05 was used to control for false positive activations. To assess the amplitude of activity, ROIs in the left and right pallidum were defined *a priori* using the AAL atlas based on previous work of Kraft et al. [11] and Feigin et al. [59] revealing the activity in BG in patients on and off medication. Then, PSC was calculated for each level of experimental factors using the same procedure as described above. Similarly, pairwise statistical comparisons were evaluated between the particular modeling approaches for the levodopa ON condition. Since BG were not activated in the levodopa OFF medication condition at all (time courses corresponded to noise), we decided not to perform any statistics for that condition.

## Results

Comparing OFF and ON conditions, the UPDRS-III scores dropped significantly (*F*(1,11) = 122.52, *p*<0.001) from 33.5 (9.0) to 9.6 (4.0) (mean value with standard deviation), demonstrating the improvement of patients' motor symptoms in the ON condition. Analysis of lateralized hemibody UPDRS-III scores in the OFF condition showed non-significant left/right asymmetry suggesting that patients with main involvement of the right or left hemispheres were represented equally in our study.

**Figure 2:**
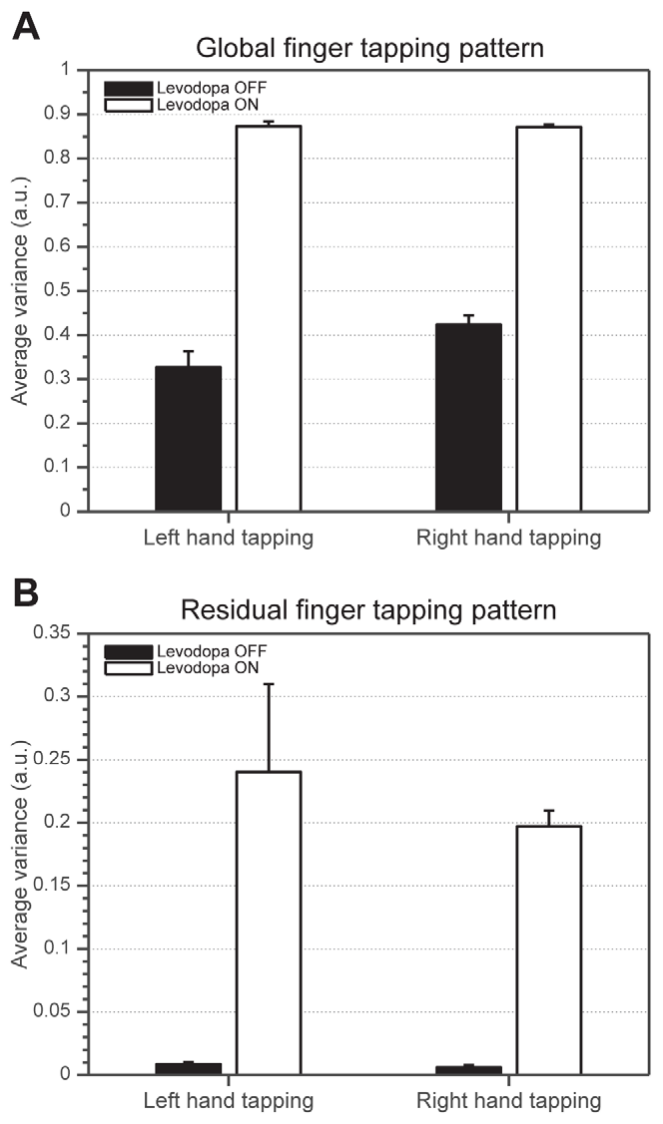
Variability of movement on behavioral level. Each bar represents average variance of movement performance calculated from a collection of measurement sessions separated for each level of experimental factors ‘Hand’ and ‘Levodopa medication’; displayed as mean+standard error. A: Average variances of global movement pattern representing the most coherent parts of glove recordings – finger tapping itself. Main effect of ‘Levodopa medication’ is significant (*p*<0.001) and an interaction between ‘Levodopa medication’ and ‘Hand’ is significant (*p*<0.05). B: Average variances of residual movement pattern representing stochastic, variable quantity of participants' motor behavior. Main effect of ‘Levodopa medication’ is significant (*p*<0.001). a.u. - arbitrary unit.

Behavioral analyses of the hand revealed a significant increase in the movement variability in the ON condition compared to OFF in both global (*F*(1,11) = 14.83, *p*<0.001) and residual (*F*(1,11) = 33.61, *p*<0.001) movement patterns (Figure 2). Moreover, a significant interaction between RIGHT/LEFT hand tapping and the OFF/ON medication condition was found in the global movement pattern (Figure 2; *F*(2,11) = 5.57, *p* = 0.04). The resting tremor and dyskinesias were absent in the course of the experiment in all our patients which was confirmed by analysis of the glove motion during resting periods of the task.

**Figure 3:**
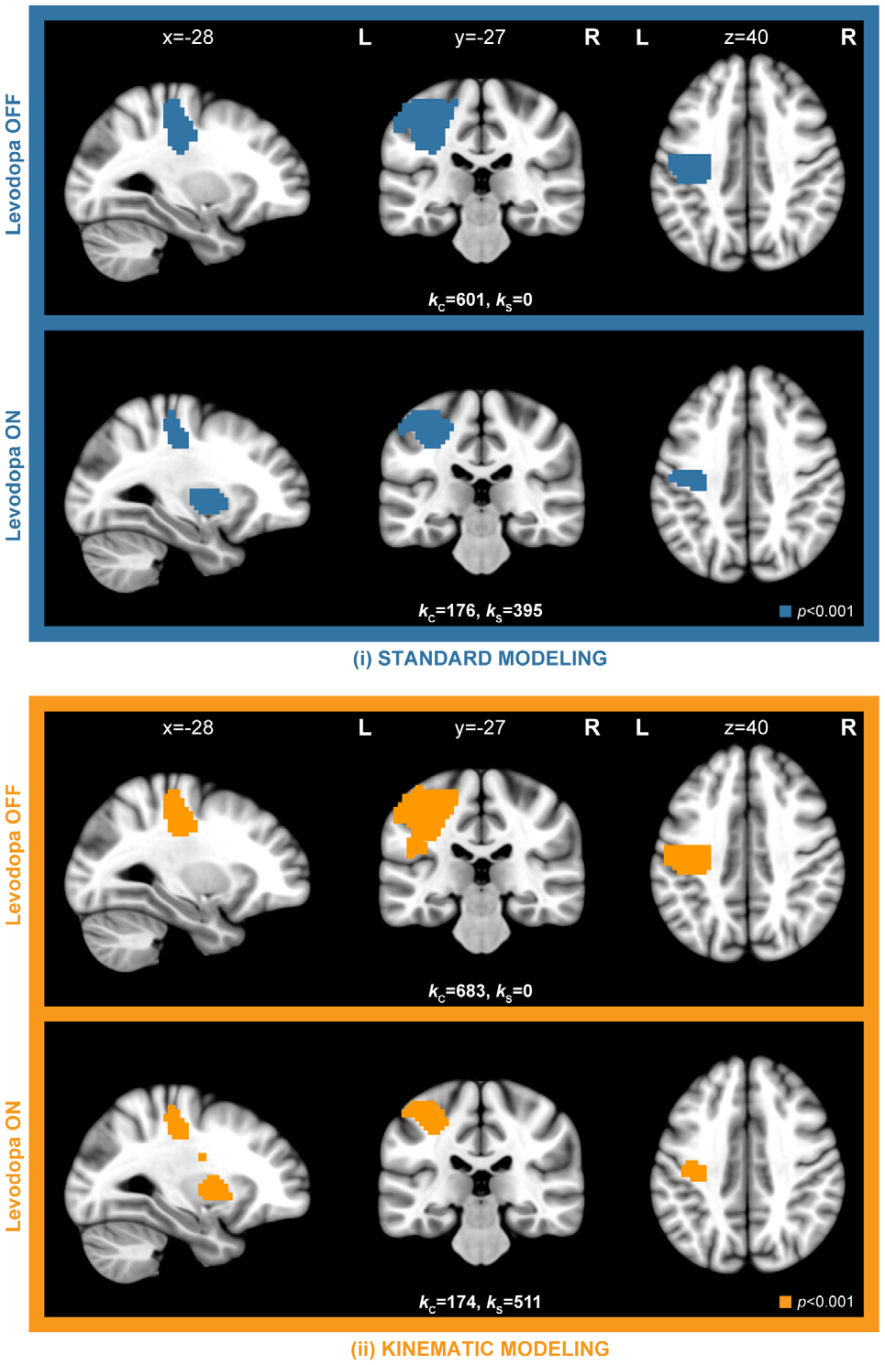
Random-effects parametric maps showing brain correlates of right finger movements with and without dopaminergic medication. Maps were obtained by separate analysis (one sample *t*-test) of both medication-free (OFF) and medication (ON) conditions on the group level (top, blue: group maps obtained by standard first-level modeling without further assumptions on motor performance; bottom, orange: group maps obtained by mean kinematic modeling technique taking motor performance into account). The brain correlates of left finger movements are not shown here, nevertheless resulted in a similar activity pattern as right finger movements, with a cortical cluster located in the contralateral hemisphere. Maps were adjusted with a threshold of *p*<0.001; uncorrected and extended to 150 voxels to show only significant clusters (*p*<0.05; FWE corrected) on the cluster level. FWE - Family Wise Error. *k*_C_ - number of activated voxels in cortical cluster. *k*_S_ - number of activated voxels in subcortical cluster.

**Figure 4:**
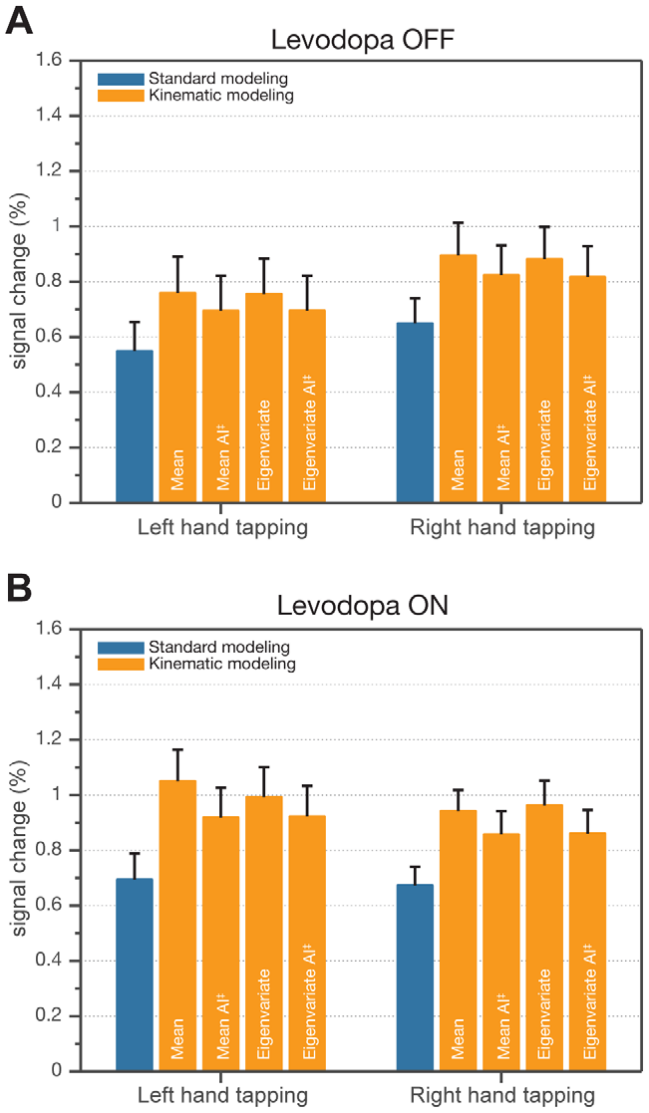
Comparison of modeling approaches as average effect size in anatomical ROI (contralateral precentral gyrus). Each bar represents the average value from ROI for the ‘group-level mean’ PSC image, and each error bar the average of the ‘group-level standard error’ PSC image. A: Percent signal change for the Levodopa OFF condition and all modeling approaches. B: Percent signal change for the Levodopa ON condition and all modeling approaches. AI^‡^ - amplitude invariant.

FMRI analyses performed for each hand and both medication conditions independently, with the purpose of showing neural correlates of finger movements, revealed brain activity in the primary motor cortex in the hemisphere contralateral to finger tapping. However, for each hand, a decreased extent of activity in the primary motor cortex was observed with levodopa intake (Figure 3). In addition, as opposed to OFF, significant activity was detected in subcortical areas (BG) after levodopa administration. Comparing OFF and ON separately, a sensitivity increase in the extent of activity using kinematic modeling compared to the standard one was found (Figure 3; see *k*_C_, *k*_S_). All kinematic approaches showed significantly higher amplitude of activity compared to the standard approach, in particular in the precentral gyrus with patients OFF (Figure 4A; Table 2) and ON (Figure 4B; Table 2) medication. Also, a significant difference between amplitude-sensitive and amplitude-invariant versions of both kinematic approaches was observed for both conditions with an exception of the eigenvariate kinematic approach in OFF condition (Table 2).

**Table 2:** Main effect of ‘Modeling approach’: percent signal change in anatomical ROI (left, right precentral gyrus) as *F*-statistics obtained by comparing particular modeling approaches in levodopa OFF and levodopa ON conditions.

Modeling approaches compared	F-statistic		Significance level	
	OFF	ON	OFF	ON
Standard – Kinematic, mean	*F*(1, 11) = 55.86	*F*(1, 11) = 108.20	*p*<0.001	*p*<0.001
Standard – Kinematic, mean AI^‡^	*F*(1, 11) = 59.77	*F*(1, 11) = 85.43	*p*<0.001	*p*<0.001
Standard – Kinematic, eigenvariate	*F*(1, 11) = 63.75	*F*(1, 11) = 68.21	*p*<0.001	*p*<0.001
Standard – Kinematic, eigenvariate AI^‡^	*F*(1, 11) = 52.97	*F*(1, 11) = 77.46	*p*<0.001	*p*<0.001
Kinematic, mean – Kinematic, mean AI^‡^	*F*(1, 11) = 23.26	*F*(1, 11) = 37.86	*p* = 0.001	*p*<0.001
Kinematic, eigenvariate – Kinematic, eigenvariate AI^‡^	*F*(1, 11) = 3.64	*F*(1, 11) = 18.15	*p* = 0.083	*p* = 0.001

AI^‡^ - amplitude invariant.

**Figure 5:**
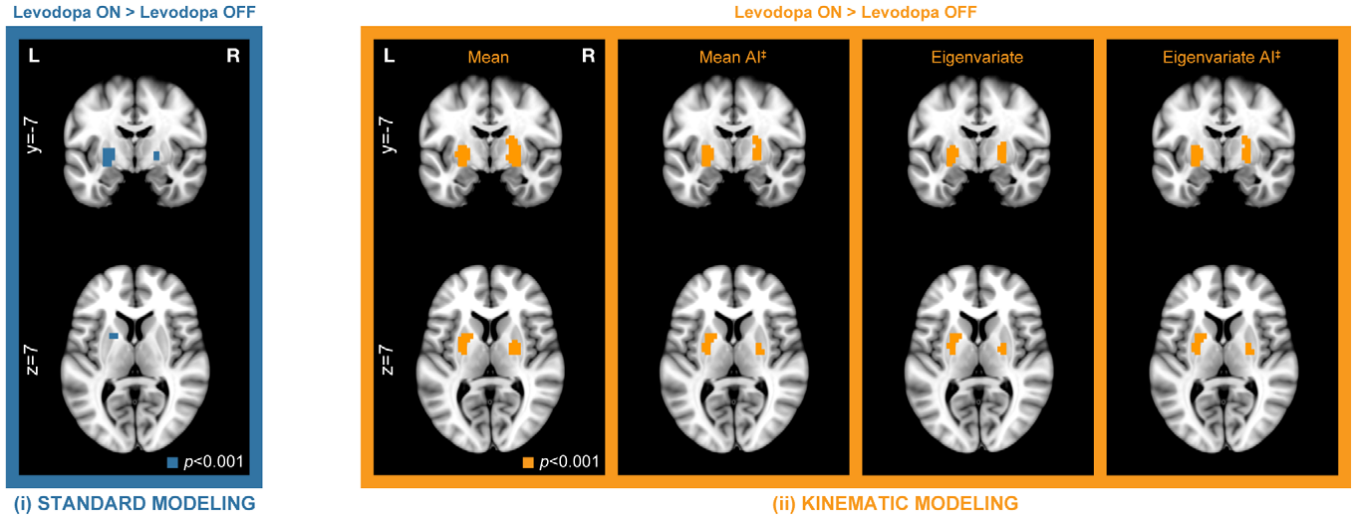
Group-level response (ON-OFF) of PD patients to levodopa treatment. Uncorrected threshold of *p*<0.001 was adopted and maps were overlaid on coronal and axial slices (left, blue: group maps obtained by standard first-level modeling without further assumptions on motor performance; right, orange: improvement of group-level maps obtained using various kinematic modeling techniques taking movement performance into account). AI^‡^ - amplitude invariant.

Investigating the difference between both medication conditions, an increased bilateral response to levodopa in the putamen and globus pallidus was revealed. Interestingly, the increased BOLD response in the ON condition was present solely within areas of BG and not observed in the primary motor cortex. This result was obtained with all modeling methods (Figure 5; *p*<0.001 uncorrected). Strikingly, all variations of kinematic modeling outperformed standard modeling and resulted in an extensive sensitivity increase, and provided a larger spatial extent of activity and higher FWE-corrected cluster *p*-values (Figure 5; Table 3). In contrast, the right subcortical cluster obtained with standard modeling did not remain significant after FWE multiple test correction.

**Table 3:** List of performance measures revealed by all modeling approaches.

Modeling approach	Cluster p-value		Number of activated voxels		Peak t-value	
	Left cluster	Right cluster	Left cluster	Right cluster	Left cluster	Right cluster
Standard	0.021	0.073	64	34	4.27	4.21
Kinematic, mean	0.017	0.007	68	91	4.37	4.59
Kinematic, mean AI^‡^	0.012	0.025	79	59	4.22	4.28
Kinematic, eigenvariate	0.010	0.014	80	71	4.20	4.75
Kinematic, eigenvariate AI^‡^	0.029	0.011	80	55	4.21	4.30

Showed items include *p*-values of clusters, numbers of activated voxels and peak *t*-values. Cluster *p*-value is FWE (Family Wise Error) corrected for multiple comparisons at *p*<0.05. Number of activated voxels are at the threshold of *p*<0.001 (uncorrected). AI^‡^ - amplitude invariant.

**Figure 6:**
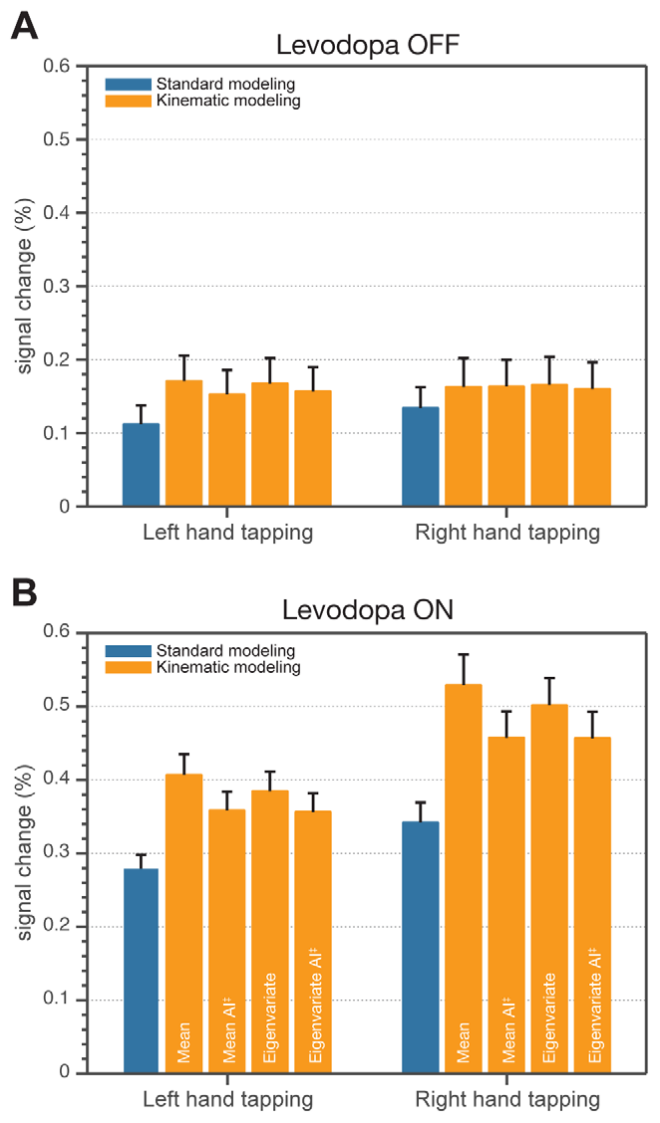
Comparison of modeling approaches as average effect size in anatomical ROI (left and right pallidum). Each bar represents the average value from ROI for the ‘group-level mean’ PSC image, and each error bar the average of the ‘group-level standard error’ PSC image. A: Percent signal change for the Levodopa OFF condition and all modeling approaches. In contrast to the ON condition, in the OFF condition, the basal ganglia were not activated so the data corresponds to noise. B: Percent signal change for the Levodopa ON condition and all modeling approaches. AI^‡^ - amplitude invariant.

Effect sizes represented as percent signal change in ROIs located in the left and right pallidum revealed differences between standard and kinematic modeling approaches even without taking inter-individual differences into account (Figure 6B). With regard to inter-individual variability, rmANOVA showed significance in the main effect of ‘Modeling approach’ (*p*<0.05) with all kinematic approaches. Additionally, a significant difference (*p*<0.05) between amplitude-sensitive and amplitude-invariant versions of both (mean, eigenvariate) kinematic approaches was discovered (Figure 6B). Table 4 summarizes the results in more detail.

**Table 4:** Main effect of ‘Modeling approach’: percent signal change in anatomical ROI (left and right pallidum) as *F*-statistics obtained by comparing particular modeling approaches in levodopa ON condition.

Modeling approaches compared	F-statistic	Significance level
Standard – Kinematic, mean	*F*(1, 11) = 14.83	*p* = 0.003
Standard – Kinematic, mean AI^‡^	*F*(1, 11) = 13.12	*p* = 0.003
Standard – Kinematic, eigenvariate	*F*(1, 11) = 17.29	*p* = 0.002
Standard – Kinematic, eigenvariate AI^‡^	*F*(1, 11) = 13.28	*p* = 0.004
Kinematic, mean – Kinematic, mean AI^‡^	*F*(1, 11) = 5.58	*p* = 0.038
Kinematic, eigenvariate – Kinematic, eigenvariate AI^‡^	*F*(1, 11) = 6.24	*p* = 0.030

AI^‡^ - amplitude invariant.

## Discussion

We investigated the benefit of controlling PD patients' movement within a finger-tapping fMRI experiment by taking the movement parameters into account in the analysis. Our results provide clear evidence of increasing sensitivity in detecting brain activity in PD patients using fMRI analyses considering on-line quantification of their motor outcome, compared to generic fMRI statistics.

Solely behavioral analyses revealed substantial differences in motor outcome of PD patients between two experimental manipulations requiring repeated sessions, underlining the importance of controlling for movement to obtain ‘true’ brain motor responses with fMRI.

Previous studies investigating levodopa intervention in PD patients with fMRI have produced conflicting results [17], [20], [60]. Contradictory activity patterns solely in cortical areas such as the supplementary motor area (SMA), premotor cortex (PMC) and primary motor cortex (M1) were observed. Surprisingly, no activity was detected in subcortical areas such as BG – areas closely associated with PD – possibly due to a lack of statistical power with a combination of relatively subtle BOLD responses in those areas [31]. A more recent study by Ng et al. [18] discussing conflicting results and interpretations in previous studies concluded that they only investigated the amplitude of BOLD response and neglected the spatial pattern of levodopa-induced activity. They showed that the main effect of levodopa seems to be a spatial ‘focusing effect’ in both subcortical and cortical structures. Work by Kraft et al. [11] exploited a bimanual task to increase BOLD responses in BG and showed a bilateral striatal activity in PD patients as a response to levodopa treatment. However, only Haslinger et al. [60] explicitly applied the behavioral motor information in fMRI modeling. In our opinion, not taking the movement into account in HR modeling may have been a sufficient source of bias in interpretations in the aforementioned studies, considering the close relationship between patients' motor performance and dopaminergic medication. Using flexible-factorial random-effects design, we increased the statistical power by joining data from left and right finger movements in one model. We observed a significant increase in the BOLD response in BG as a result of levodopa intake in PD patients compared to the medication-free condition, which is in agreement with the fMRI results of Kraft et al. [11] and PET results of Feigin et al. [59]. This supports the idea of a ‘normalizing effect’ of levodopa in putamen in cortico-subcortico-cortical circuits of the current pathophysiological model [1], [61]. The response in BG nevertheless appears to a different degree of amplitude and extent using either standard or kinematic modeling techniques.

In this study, kinematic modeling was performed using the behavioral information incorporated in a single regressor, in order to preserve the design efficiency and avoid covariate correlation problems because the movement-related regressor is likely to correlate with the stimulus-based regressor to a high degree. In the interpretations of fMRI statistical tests, correlation is a potential source of ambiguity arising even in the simplest models [62]. Orthogonalizing the regressor in respect to other as a method to tackle correlation problems in studies controlling for motor performance of participants [33]–[35] is an option; however, it may not solve the problem because of decreased sensitivity. The type of modeling framework used in this work is especially useful for identifying ‘true’ motor brain responses. Extending the design with another stimulus-specific regressor would be particularly interesting for investigating phenomena such as neural correlates of motor planning and preparation, sensorimotor integration, or identifying motor circuitry responsible for pathological movement. In such cases, the model must incorporate experiment information in order to discriminate between actual movement and stimuli presentation.

We used two types of linear Gaussian models to input glove recordings with hemodynamic modeling and compared this approach with generic analysis commonly used in PD motor studies, where no quantitative analysis of movement performance is usually reported. In investigating motor abnormalities with task-related fMRI, experimental set-up and timing do not provide sufficient information for modeling the hemodynamic response adequately, resulting in sensitivity decrease. All formerly described kinematic models yielded better fits than the standard analysis. We favor the eigenvariate approach as being theoretically more sensitive in reducing the dimensionality of high-dimensional data as compared to simple averaging. It preserves the dominant task-related movement pattern by assigning higher weights to inputs contributing to it while eliminating components likely corresponding to noise. On the other hand, the mean model accounts for every single input (sensor) to the same degree, which may result in detecting movement deviations specific to the disease, with the penalty of a higher probability for introducing noise. In fact, both approaches are often correlated and result in a similar outcome, especially if the input data are pre-smoothed (i.e., low-pass filtered). Then the input variables have a higher likelihood of co-varying and reflect to similar extents on the first principal component, which roughly speaking, is a process of averaging. Both of the approaches presented provided a clear increase in sensitivity and we recommend them both for merging multi-dimensional movement recordings such as those presented here.

A clear sensitivity increase was gained by using all variations of kinematic approaches, as they accounted for undesired movement variations of participants in hemodynamic modeling. This increased the sensitivity in detecting correlations of kinematic models' predictors with measured brain responses and contributed to the reduction of ‘type II’ errors on the individual level. With the repetitive nature of the experiment and the experimental intervention altering the motor outcome significantly, an additive character of the sensitivity increase was also demonstrated on the group level by treating both conditions of dopaminergic treatment separately and by contrasting their difference. Besides conspicuous and dominant increase of sensitivity caused by accurate timing of the HRF, a beneficial effect of considering the amplitude of movement using amplitude-sensitive kinematic approaches in forming the HRF is also evident. Waldvogel et al. [26] stated that an increased neuronal firing rate resulting from healthy subjects tapping with a larger amplitude leads to higher synaptic activity, higher metabolic demand, and therefore an increased BOLD signal. Their alternative explanation is based on the fact that the observed BOLD increase is caused by subjects using additional muscles needed to stabilize the hand during large-amplitude movements. However, they did not provide an account of the quantitative reciprocal relationship between the two. With one exception (eigenvariate kinematic approach, levodopa OFF), we detected significant increases of percent signal change using amplitude-sensitive versions compared to amplitude-invariant versions of both linear Gaussian kinematic approaches. Considering the conclusions by Waldvogel et al. [26] and our results, we conclude that there is a mutual relationship between an increase in movement amplitude and HR in PD patients, too. In addition to ensuring that timing is correct, amplitude of movement in PD fMRI motor experiments must also be controlled.

### Conclusions

The approach presented here used an fMRI design of alternating movement/rest blocks, but might be also suitable for event-related designs. In comparison to block designs, event-related designs require more variable tasks in terms of motor performance (to preserve the design efficient) and where a precise knowledge of behavioral information such as subjects' motor performance is of greater importance according to numerical simulations by MacIntosh et al. [33]. In PD patients, with increasing demands and task difficulty, and with a wide spectrum of possible experimental manipulations such as medication, without controlling for movement, one can barely detect undesirable movement deviations. Our results demonstrate the importance of controlling movements when investigating PD patients using fMRI. We strongly advocate quantitatively controlling for motor performance in order to increase the sensitivity by taking the patient's behavior into account.

## Supporting Information

Table S1 UPDRS-III* - Motor part of Unified Parkinson's Disease Rating Scale. G: gender. FS: First signs of the disease at age (years). LC: Late complications of the disease at age (years). LT: duration of levodopa treatment (years). OFF: without levodopa medication. ON: with levodopa medication.
(DOC)
