# Supplementary material for: Accounting for Movement Increases Sensitivity in Detecting Brain Activity in Parkinson's Disease
Source: PLoS One. 2012 May 1;7(5):e36271. doi: 10.1371/journal.pone.0036271 (PMC3341369; doi:10.1371/journal.pone.0036271)
Supplement: Table S1 — UPDRS-III* - Motor part of Unified Parkinson's Disease Rating Scale. G: gender. FS: First signs of the disease at age (years). LC: Late complications of the disease at age (years). LT: duration of levodopa treatment (years). OFF: without levodopa medication. ON: with levodopa medication. (DOC) [file pone.0036271.s001.doc]

Table S1. Clinical details of investigated patients.

|  |  |  |  |  |  | UPDRS-III* | | | | | | | | | | |
| --- | --- | --- | --- | --- | --- | --- | --- | --- | --- | --- | --- | --- | --- | --- | --- | --- |
|  |  |  |  |  |  | Total | | | Akinesia | | Rigidity | | Tremor | | Axial | |
| ID | Age | G | FS | LC | LT | OFF | | ON | OFF | ON | OFF | ON | OFF | ON | OFF | ON |
| 1 | 63 | M | 48 | 51 | 13 | 21 | 5 | | 7 | 2 | 8 | 2 | 2 | 0 | 4 | 1 |
| 2 | 53 | M | 42 | 50 | 7 | 45 | 9 | | 27 | 3 | 8 | 2 | 2 | 2 | 8 | 2 |
| 3 | 46 | M | 31 | 37 | 12 | 40 | 12.5 | | 22 | 8.5 | 9 | 1 | 0 | 0 | 9 | 3 |
| 4 | 64 | M | 50 | 60 | 13 | 31 | 1.5 | | 17 | 0.5 | 4 | 0 | 6 | 0 | 4 | 1 |
| 5 | 58 | M | 47 | 55 | 8 | 25.5 | 11 | | 11.5 | 4.5 | 8 | 2 | 2 | 2 | 4 | 2.5 |
| 6 | 49 | M | 40 | 47 | 5 | 20.5 | 9 | | 14.5 | 6.5 | 2.5 | 0.5 | 1 | 0.5 | 2.5 | 1.5 |
| 7 | 64 | M | 50 | 59 | 13 | 37 | 11 | | 22 | 9 | 7 | 1 | 3 | 0 | 5 | 1 |
| 8 | 53 | M | 41 | 48 | 10 | 36.5 | 10.5 | | 21 | 7 | 7 | 1 | 6 | 1 | 2.5 | 1.5 |
| 9 | 59 | M | 50 | 57 | 8 | 26 | 5.5 | | 16 | 2 | 6 | 1.5 | 0 | 0 | 4 | 2 |
| 10 | 45 | M | 31 | 39 | 6 | 47 | 20.5 | | 21.5 | 9.5 | 14 | 5 | 2.5 | 2 | 9 | 4 |
| 11 | 64 | M | 51 | 58 | 8 | 31 | 10 | | 13 | 3 | 8 | 2 | 5 | 2 | 5 | 3 |
| 12 | 53 | M | 41 | 50 | 9 | 43 | 9.5 | | 22 | 3.5 | 11 | 1 | 2 | 2 | 8 | 3 |
